# Supplementary material for: Effectiveness and safety of an absorbable modified polymer starch powder hemostat versus usual care in gynecology procedures: A prospective, multi-center, and randomized study
Source: PLoS One. 2025 Sep 11;20(9):e0331376. doi: 10.1371/journal.pone.0331376 (PMC12425258; doi:10.1371/journal.pone.0331376)
Supplement: S1 Table — (DOCX) [file pone.0331376.s001.docx]

S1 Table. Sites of application of the absorbable modified polymer (AMP) starch powder hemostat.

| **Site of Application** | **N (%)** |
| --- | --- |
| Pelvis | 32 (72.7) |
| Vaginal Dome | 4 (9.1) |
| Ovary | 3 (6.8) |
| Uterine Scar | 2 (4.6) |
| Liver | 1 (2.3) |
| Pararectal | 1 (2.3) |
| Not Reported | 1 (2.3) |
