# Supplementary material for: Effectiveness and safety of an absorbable modified polymer starch powder hemostat versus usual care in gynecology procedures: A prospective, multi-center, and randomized study
Source: PLoS One. 2025 Sep 11;20(9):e0331376. doi: 10.1371/journal.pone.0331376 (PMC12425258; doi:10.1371/journal.pone.0331376)
Supplement: S2 Table — (DOCX) [file pone.0331376.s002.docx]

S2 Table. Overview of the different non-serious adverse events reported in the study.

|  | **Events (Subjects, %)** | | | | | |
| --- | --- | --- | --- | --- | --- | --- |
|  | **All AEs** | | | **Device-related AEs** | | |
| **AE Category** | **Overall (n=90)** | **AMP (n=44)** | **SC (n=46)** | **Overall (n=90)** | **AMP (n=44)** | **SC (n=46)** |
| Abdominal wound infection | 1 (1, 1.1%) | 0 (0, 0.0%) | 1(1, 2.2%) | 0 (0, 0.0%) | 0 (0, 0.0%) | 0 (0, 0.0%) |
| Allergic reaction to  contrast during CAT | 1 (1, 1.1%) | 1 (1, 2.3%) | 0 (0, 0.0%) | 0 (0, 0.0%) | 0 (0, 0.0%) | 0 (0, 0.0%) |
| Anemia | 2 (2, 2.2%) | 1 (1, 2.3%) | 1(1, 2.2%) | 0 (0, 0.0%) | 0 (0, 0.0%) | 0 (0, 0.0%) |
| Asymptomatic pulmonary embolism  (incidental finding) | 1 (1, 1.1%) | 0 (0, 0.0%) | 1(1, 2.2%) | 0 (0, 0.0%) | 0 (0, 0.0%) | 0 (0, 0.0%) |
| Cupula hematoma  and urinary infection | 1 (1, 1.1%) | 1 (1, 2.3%) | 0 (0, 0.0%) | 1 (1, 1.1%) | 1 (1, 2.3%) | 0 (0, 0.0%) |
| Douglas hematoma | 1 (1, 1.1%) | 1 (1, 2.3%) | 0 (0, 0.0%) | 1 (1, 1.1%) | 1 (1, 2.3%) | 0 (0, 0.0%) |
| Fever and syncope (runny nose) | 1 (1, 1.1%) | 1 (1, 2.3%) | 0 (0, 0.0%) | 0 (0, 0.0%) | 0 (0, 0.0%) | 0 (0, 0.0%) |
| Headache | 1 (1, 1.1%) | 0 (0, 0.0%) | 1(1, 2.2%) | 0 (0, 0.0%) | 0 (0, 0.0%) | 0 (0, 0.0%) |
| Headache episodes | 1 (1, 1.1%) | 1 (1, 2.3%) | 0 (0, 0.0%) | 0 (0, 0.0%) | 0 (0, 0.0%) | 0 (0, 0.0%) |
| Hyperglycemia | 1 (1, 1.1%) | 0 (0, 0.0%) | 1(1, 2.2%) | 0 (0, 0.0%) | 0 (0, 0.0%) | 0 (0, 0.0%) |
| Right external iliac vein injury | 1 (1, 1.1%) | 0 (0, 0.0%) | 1(1, 2.2%) | 0 (0, 0.0%) | 0 (0, 0.0%) | 0 (0, 0.0%) |
| Sepsis (*E. coli*) | 1 (1, 1.1%) | 0 (0, 0.0%) | 1(1, 2.2%) | 0 (0, 0.0%) | 0 (0, 0.0%) | 0 (0, 0.0%) |
| Surgical scar infection | 1 (1, 1.1%) | 0 (0, 0.0%) | 1(1, 2.2%) | 0 (0, 0.0%) | 0 (0, 0.0%) | 0 (0, 0.0%) |
| DVT and GE | 1 (1, 1.1%) | 0 (0, 0.0%) | 1(1, 2.2%) | 0 (0, 0.0%) | 0 (0, 0.0%) | 0 (0, 0.0%) |
| Urinary infection | 4 (4, 4.4%) | 1 (1, 2.3%) | 3 (3, 6.5%) | 0 (0, 0.0%) | 0 (0, 0.0%) | 0 (0, 0.0%) |
| Urinary infection and  pielometritis | 1 (1, 1.1%) | 1 (1, 2.3%) | 0 (0, 0.0%) | 0 (0, 0.0%) | 0 (0, 0.0%) | 0 (0, 0.0%) |
| Uterine perforation | 1 (1, 1.1%) | 0 (0, 0.0%) | 1(1, 2.2%) | 0 (0, 0.0%) | 0 (0, 0.0%) | 0 (0, 0.0%) |
| Vaginal cuff hematoma | 1 (1, 1.1%) | 1 (1, 2.3%) | 0 (0, 0.0%) | 1 (1, 1.1%) | 1 (1, 2.3%) | 0 (0, 0.0%) |
| Vaginal spotting /  pelvic hematoma | 1 (1, 1.1%) | 1 (1, 2.3%) | 0 (0, 0.0%) | 0 (0, 0.0%) | 0 (0, 0.0%) | 0 (0, 0.0%) |
| Vaginosis By *Gardnerella* sp. | 1 (1, 1.1%) | 0 (0, 0.0%) | 1(1, 2.2%) | 0 (0, 0.0%) | 0 (0, 0.0%) | 0 (0, 0.0%) |

AEs: Adverse events; AMP: Absorbable modified polymer starch powder hemostat; SC: Standard care; CAT: Computerized axial tomography; DVT: Deep venous thrombosis; GE: Generalized embolization.
