# Supplementary material for: Effectiveness and safety of an absorbable modified polymer starch powder hemostat versus usual care in gynecology procedures: A prospective, multi-center, and randomized study
Source: PLoS One. 2025 Sep 11;20(9):e0331376. doi: 10.1371/journal.pone.0331376 (PMC12425258; doi:10.1371/journal.pone.0331376)
Supplement: S3 Table — (DOCX) [file pone.0331376.s003.docx]

S3 Table. Overview of the Serious adverse events documented over the study follow-up.

|  | **Events (Subjects, %)** | | | | | |
| --- | --- | --- | --- | --- | --- | --- |
|  | **All AEs** | | | **Device-related AEs** | | |
| **AE Category** | **Overall (n=90)** | **AMP (n=44)** | **SC (n=46)** | **Overall (n=90)** | **AMP (n=44)** | **SC (n=46)** |
| Paralytic ileus | 1 (1, 1.1%) | 0 (0, 0.0%) | 1(1, 2.2%) | 0 (0, 0.0%) | 0 (0, 0.0%) | 0 (0, 0.0%) |
| Pelvic hematoma infection | 1 (1, 1.1%) | 1 (1, 2.3%) | 0 (0, 0.0%) | 0 (0, 0.0%) | 0 (0, 0.0%) | 0 (0, 0.0%) |
| Pleural effusion and dyspnea | 2 (2, 2.2%) | 0 (0, 0.0%) | 2 (2, 4.3%) | 0 (0, 0.0%) | 0 (0, 0.0%) | 0 (0, 0.0%) |
| Retroperitoneal abscess | 1 (1, 1.1%) | 1 (1, 2.3%) | 0 (0, 0.0%) | 1 (1, 1.1%) | 1 (1, 2.3%) | 0 (0, 0.0%) |

AMP: Absorbable modified polymer starch powder hemostat; SC: Standard care; AE: Adverse event.
