# Supplementary material for: Effectiveness and safety of an absorbable modified polymer starch powder hemostat versus usual care in gynecology procedures: A prospective, multi-center, and randomized study
Source: PLoS One. 2025 Sep 11;20(9):e0331376. doi: 10.1371/journal.pone.0331376 (PMC12425258; doi:10.1371/journal.pone.0331376)
Supplement: S4 Table — (DOCX) [file pone.0331376.s004.docx]

S4 Table. Time of onset and evolution of the different serious adverse events reported during the study.

| **Patient** | **Visit** | **Group** | **SAE** | **Action taken** |
| --- | --- | --- | --- | --- |
| Case 1 | Post- Operative | SC | Paralytic ileus | Resolved with medication |
| Case 2 | Other | SC | Pleural effusion and dyspnea | Surgical drainage |
| Case 3 | 30-day Follow-Up | SC | Pleural effusion and dyspnea | Pleural drainage |
| Case 4 | Other | AMP | Pelvic hematoma infection | Resolved with antibiotics |
| Case 5 | Other | AMP | Retroperitoneal abscess | Recovered with drainage |

AMP: Absorbable modified polymer starch powder hemostat; SC: Standard care; SAE: Serious adverse event.
