# Supplementary material for: Effectiveness and safety of an absorbable modified polymer starch powder hemostat versus usual care in gynecology procedures: A prospective, multi-center, and randomized study
Source: PLoS One. 2025 Sep 11;20(9):e0331376. doi: 10.1371/journal.pone.0331376 (PMC12425258; doi:10.1371/journal.pone.0331376)
Supplement: Attachment — Submitted filename: Rebuttal letter PerClot PlosOne R1 1.1.docx [file pone.0331376.s006.docx]

Response to the reviewers, manuscript PONE-D-25-02293, entitled

“Effectiveness and safety of an absorbable modified polymer starch powder hemostat versus usual care in gynecology procedures: A prospective, multi-center, and randomized study”.

Dear Editor-in-Chief, I have considered the comments made by the reviewers and I hope that the paper improves greatly thanks to these comments.

I am sending you the revised manuscript and the rebuttal letter providing a point-by-point response to each of the numbered reviewer comments.

**#Journal requirements:**

**When submitting your revision, we need you to address these additional requirements.**

**1. Please ensure that your manuscript meets PLOS ONE's style requirements, including those for file naming. The PLOS ONE style templates can be found at**

<https://journals.plos.org/plosone/s/file?id=wjVg/PLOSOne_formatting_sample_main_body.pdf> **and**

[https://journals.plos.org/plosone/s/file?id=ba62/PLOSOne_formatting_sample_title_authors_affiliations.pdf](https://track.editorialmanager.com/CL0/https:%2F%2Fjournals.plos.org%2Fplosone%2Fs%2Ffile%3Fid=ba62%2FPLOSOne_formatting_sample_title_authors_affiliations.pdf/1/010f0195b98476ea-a41048bc-d1ca-48d8-8666-a6acf5e01a38-000000/_pe9x52ThkpgzrLkc_ePxwY9bpr7SZzO9ZkZu2pSzz4=203).

**2. We note that the grant information you provided in the ‘Funding Information’ and ‘Financial Disclosure’ sections do not match.**

**When you resubmit, please ensure that you provide the correct grant numbers for the awards you received for your study in the ‘Funding Information’ section.**

It was corrected.

No specific numbers have been assigned to the Grants received.

**3. Thank you for stating the following financial disclosure:**

**[Cryolife, Europe**

**Baxter (Founded Medical writing)].**

**Please state what role the funders took in the study. If the funders had no role, please state: ""The funders had no role in study design, data collection and analysis, decision to publish, or preparation of the manuscript.""**

**If this statement is not correct you must amend it as needed.**

**Please include this amended Role of Funder statement in your cover letter; we will change the online submission form on your behalf.**

Thank you very much indeed for the comment, we highly appreciate it.

The following sentence was added in the Funding section and in the cover letter: “Cryolife was involved in study funding, study design, provision of study materials, data collection and analysis”.

“Baxter was involved in medical writing funding, preparation of the manuscript, decision to publish”.

**4. Thank you for stating the following in the Competing Interests section:**

**[Barbara Costantini, Jordi Ponce and Giovanni Scambia served as speakers for Baxter and have received speaker honoraria according to fair market value.].**

**Please confirm that this does not alter your adherence to all PLOS ONE policies on sharing data and materials, by including the following statement: ""This does not alter our adherence to  PLOS ONE policies on sharing data and materials.” (as detailed online in our guide for authors** [**http://journals.plos.org/plosone/s/competing-interests**](http://journals.plos.org/plosone/s/competing-interests)**).  If there are restrictions on sharing of data and/or materials, please state these. Please note that we cannot proceed with consideration of your article until this information has been declared.**

**Please include your updated Competing Interests statement in your cover letter; we will change the online submission form on your behalf.**

The following sentence was added in the “Conflict of Interests” section and in the cover letter: “This does not alter our adherence to PLOS ONE policies on sharing data and materials.”

**5. We note that you have indicated that there are restrictions to data sharing for this study. For studies involving human research participant data or other sensitive data, we encourage authors to share de-identified or anonymized data. However, when data cannot be publicly shared for ethical reasons, we allow authors to make their data sets available upon request. For information on unacceptable data access restrictions, please see** [**http://journals.plos.org/plosone/s/data-availability#loc-unacceptable-data-access-restrictions**](http://journals.plos.org/plosone/s/data-availability#loc-unacceptable-data-access-restrictions)**.**

**Before we proceed with your manuscript, please address the following prompts:**

**a) If there are ethical or legal restrictions on sharing a de-identified data set, please explain them in detail (e.g., data contain potentially identifying or sensitive patient information, data are owned by a third-party organization, etc.) and who has imposed them (e.g., a Research Ethics Committee or Institutional Review Board, etc.). Please also provide contact information for a data access committee, ethics committee, or other institutional body to which data requests may be sent.**

**b) If there are no restrictions, please upload the minimal anonymized data set necessary to replicate your study findings to a stable, public repository and provide us with the relevant URLs, DOIs, or accession numbers. Please see** [**http://www.bmj.com/content/340/bmj.c181.long**](http://www.bmj.com/content/340/bmj.c181.long) **for guidelines on how to de-identify and prepare clinical data for publication. For a list of recommended repositories, please see** [**https://journals.plos.org/plosone/s/recommended-repositories**](https://journals.plos.org/plosone/s/recommended-repositories)**. You also have the option of uploading the data as Supporting Information files, but we would recommend depositing data directly to a data repository if possible.**

**Please update your Data Availability statement in the submission form accordingly.**

The following sentence was added in the Data Availability Statement: “Our study utilizes third-party data that are the property of Artivion, formerly known as CryoLife Europa. As these data are proprietary, they cannot be publicly shared. However, interested researchers may contact Artivion directly to inquire about potential access to the data (https://artivion.com/; 1655 Roberts Blvd., NW; Kennesaw, GA 30144 USA.”.

**6.  Please include captions for your Supporting Information files at the end of your manuscript, and update any in-text citations to match accordingly. Please see our Supporting Information guidelines for more information:** [**http://journals.plos.org/plosone/s/supporting-information**](http://journals.plos.org/plosone/s/supporting-information)**.**

It was corrected.

**Reviewers' comments:**

**Reviewer's Responses to Questions**

**Comments to the Author**

**1. Is the manuscript technically sound, and do the data support the conclusions?**

**The manuscript must describe a technically sound piece of scientific research with data that supports the conclusions. Experiments must have been conducted rigorously, with appropriate controls, replication, and sample sizes. The conclusions must be drawn appropriately based on the data presented.**

**Reviewer #1: No**

**Reviewer #2: Yes**

**2. Has the statistical analysis been performed appropriately and rigorously?**

**Reviewer #1: No**

**Reviewer #2: Yes**

**3. Have the authors made all data underlying the findings in their manuscript fully available?**

**The PLOS Data policy requires authors to make all data underlying the findings described in their manuscript fully available without restriction, with rare exception (please refer to the Data Availability Statement in the manuscript PDF file). The data should be provided as part of the manuscript or its supporting information, or deposited to a public repository. For example, in addition to summary statistics, the data points behind means, medians and variance measures should be available. If there are restrictions on publicly sharing data—e.g. participant privacy or use of data from a third party—those must be specified.**

**Reviewer #1: Yes**

**Reviewer #2: Yes**

**4. Is the manuscript presented in an intelligible fashion and written in standard English?**

**PLOS ONE does not copyedit accepted manuscripts, so the language in submitted articles must be clear, correct, and unambiguous. Any typographical or grammatical errors should be corrected at revision, so please note any specific errors here.**

**Reviewer #1: Yes**

**Reviewer #2: Yes**

**5. Review Comments to the Author**

**Please use the space provided to explain your answers to the questions above. You may also include additional comments for the author, including concerns about dual publication, research ethics, or publication ethics. (Please upload your review as an attachment if it exceeds 20,000 characters).**

**Reviewer #1:**

**1. The study lacks a statistically valid sample size justification. That could not be found in the protocol, either. The paper mentions that 90 subjects are needed as per the protocol. How was that calculated? This reviewer found no valid statistical design section in the protocol unless it is under another section. One should not have to search for this information. Section 9.0 (Statistical Considerations) of the protocol is totally inadequate. All it mentions is an ITT and per protocol strategy. Where or what was the intended non inferiority margin? Since non inferiority was mentioned in the manuscript, it should have been incorporated into the design of the study.**

Thank you very much indeed for the comment, we highly appreciate it.

We fully agree with the reviewer about the limitation of the lack of statistically valid sample size justification. This was added as a study limitation.

The current study was a Phase IV, observational (post-market) study designed to provide descriptive and exploratory data rather than confirmatory evidence. As stated in the study protocol, the sample size was not determined to achieve a specific statistical power to detect differences between the PerClot and Standard of Care (SoC) patient populations. Instead, this study followed an observational, case-controlled design, in which patients were grouped based on treatment exposure rather than through true randomization, which is characteristic of experimental studies such as randomized controlled trials (RCTs). The PerClot group represented the case study cohort, while the SoC group served as the control, allowing for comparative assessment of specific clinical characteristics between the two cohorts.

Given the exploratory nature of this study, our primary objective was to gather preliminary evidence rather than to definitively establish non-inferiority. Although prospective sample size calculation is the preferred methodological approach, as the reviewer correctly noted, a post-hoc analysis was conducted to estimate the number of patients required to detect significant differences based on the observed results. This analysis provided valuable insights into the potential adequacy of the study’s sample size, reinforcing the descriptive and hypothesis-generating role of this investigation.

The following paragraph was added as a study limitation: “Another important limitation of this study is the lack of a statistically valid sample size justification. While a prospective sample size calculation would have been the ideal approach, a post-hoc analysis was performed to estimate the number of patients required to detect significant differences based on the observed results. With an alpha risk of 0.05 in a two-sided test and sample sizes of 44 patients in the AMP group and 46 in the SC group, the statistical power to detect the observed differences as significant was only 16%. Furthermore, to achieve a power of 80% with an alpha of 0.05, a total of 353 patients per group would have been required for the observed differences in the proportion of patients achieving hemostasis within 10 minutes to reach statistical significance”.

**2. Apparently the ITT and per protocol results compare as they are the same as noted in Table 3. However, even though the groups look fairly balanced was there any attempt to look at covariate association for achieving hemostasis along with treatment in a multivariate setting such as the logistic regression? The investigation does seem incomplete, unless other possible associative factors with hemostasis are examined in a more rigorous statistical setting. The statistical handling of this data (design and analysis) is obviously incomplete.**

Thank you for your insightful comment. We fully acknowledge the importance of examining potential covariate associations with hemostasis in a more rigorous statistical framework, such as logistic regression, to strengthen the analysis. Unfortunately, we do not have access to the original dataset but only to the statistical report provided by the study promoter, which limits our ability to perform additional multivariate analyses. Consequently, we are unable to explore potential confounding factors that may influence hemostasis outcomes. While our study primarily focused on comparing ITT and per-protocol results, we recognize that a more comprehensive statistical approach could provide further insights. We appreciate your valuable suggestion and acknowledge this as a limitation of our study.

The following sentence was added as a limitation of the study: “Additionally, the absence of a multivariate analysis to assess potential covariate associations with hemostasis, which could have offered a more detailed statistical evaluation, may be considered a limitation of the study”.

**Reviewer #2:**

**I found the manuscript, to be of high quality. The methodology is sound, the data are presented clearly, and the discussion is insightful. The authors have effectively addressed the research question, and the article is well-designed for publication.**

Thank you very much indeed for this insightful comment; we truly appreciate it.
