# Supplementary material for: Effectiveness and safety of an absorbable modified polymer starch powder hemostat versus usual care in gynecology procedures: A prospective, multi-center, and randomized study
Source: PLoS One. 2025 Sep 11;20(9):e0331376. doi: 10.1371/journal.pone.0331376 (PMC12425258; doi:10.1371/journal.pone.0331376)
Supplement: Attachment — Submitted filename: Rebuttal letter PerClot PlosOne R2 1.1.docx [file pone.0331376.s007.docx]

Response to the reviewers, manuscript PONE-D-25-02293R1, entitled

“Effectiveness and safety of an absorbable modified polymer starch powder hemostat versus usual care in gynecology procedures: A prospective, multi-center, and randomized study”.

Dear Editor-in-Chief, I have considered the comments made by the reviewers and I hope that the paper improves greatly thanks to these comments.

I am sending you the revised manuscript and the rebuttal letter providing a point-by-point response to each of the numbered reviewer comments.

**# Journal Requirements:**

**Please review your reference list to ensure that it is complete and correct. If you have cited papers that have been retracted, please include the rationale for doing so in the manuscript text, or remove these references and replace them with relevant current references. Any changes to the reference list should be mentioned in the rebuttal letter that accompanies your revised manuscript. If you need to cite a retracted article, indicate the article’s retracted status in the References list and also include a citation and full reference for the retraction notice.**

Reference list has been reviewed.

**# Review Comments to the Author
Please use the space provided to explain your answers to the questions above. You may also include additional comments for the author, including concerns about dual publication, research ethics, or publication ethics. (Please upload your review as an attachment if it exceeds 20,000 characters)**

**Reviewer #1: (No Response)**

**Reviewer #3: In this revised submission, Dr. Garcia-Tejedor and colleagues compare the use of absorbable modified polymer starch powder hemostat (AMP-SPH) compared to standard of care (SC). The submission could be improved with respect to technique, data, and statistics by the following:**

**1. The bleeding severity descriptions provided suggest that AMP-SPH may have been used outside of its approved labeling. Providing an explanation of why this was permitted would be useful.**

According to the study protocol, and the intraoperative inclusion criteria: Subject experiences generalized oozing or mild to moderate bleeding which is consistent with the requirement for a hemostatic agent. Bleeding was defined and documented in the CRF as:

- **Oozing -** The rate of bleeding is slow. This is what one may expect from capillary, venular, or arteriolar bleeding.
- **Mild bleeding -** The rate of bleeding is slightly faster than oozing. There is no pulsatile flow present. This is what one may expect from capillary, venular, or arteriolar bleeding.
- **Moderate bleeding.** There may be a weak pulsatile flow present. If there is a pulsatile flow, the rate of blood flow is similar to the rate of flow for mild bleeding. If there is no pulsatile flow, the rate of blood flow is faster than mild bleeding.
- **Severe bleeding.** For the most part, pulsatile flow is present. If there is no pulsatile flow, then the rate of blood flow is extremely rapid.

If we were to apply the Vibe Scale (Lewis KM, Li Q, Jones DS, Corrales JD, Du H, Spiess PE, Lo Menzo E, DeAnda A Jr. Development and validation of an intraoperative bleeding severity scale for use in clinical studies of hemostatic agents. Surgery. 2017 Mar;161(3):771-781. doi: 10.1016/j.surg.2016.09.022.) to these definitions for bleeding in this chart:

- Oozing - Vibe 1 >1-5ml/min
- Mild - Vibe 2 >5-10ml/min
- Moderate - Vibe 2-3
- Severe - Vibe 3 >10-50ml/min (not Vibe 4)

Except for the 1 outlier that was reported to have severe bleeding; All the patients bleeding did fall into the labeling. Probably this severe bleeding patient should have been excluded from the study as this patient is an outlier - although, this patient apparently did not meet the specific criteria to be excluded by the surgeon - according to the blood loss classification of the ATLS classification chart. The patient was not a Class II-IV.

Intraoperative Exclusion Criteria:

Subject has any major intraoperative bleeding incidences (i.e., American College of Surgeons Advanced Trauma Life Support Class II, III or IV Hemorrhage [2013])

ATLS classification of blood loss is classified into four classes based on the estimated percentage of blood volume loss. Class I involves up to 15% blood loss (750ml), Class II involves 15-30% (750-1500ml), Class III involves 30-40% (1500-2000ml), and Class IV involves >40% blood loss (>2000ml). These classifications correlate with changes in vital signs like heart rate and blood pressure. (Spahn, D.R., Bouillon, B., Cerny, V. et al. Management of bleeding and coagulopathy following major trauma: an updated European guideline. Crit Care 17, R76 (2013). https://doi.org/10.1186/cc12685).

**2. Achieving hemostasis at 10 minutes was the primary endpoint, but 10 minutes is considered long for the primary endpoint in time to hemostasis studies in the current environment. Supplying a table with the results for the degree of hemostasis at all observed time points would be helpful.**

While 10 minutes may not represent the current clinical study bleeding assessment criteria, this study was started in 2015. If we apply the Vibe bleeding scale rates (Lewis KM, Li Q, Jones DS, Corrales JD, Du H, Spiess PE, Lo Menzo E, DeAnda A Jr. Development and validation of an intraoperative bleeding severity scale for use in clinical studies of hemostatic agents. Surgery. 2017 Mar;161(3):771-781. doi: 10.1016/j.surg.2016.09.022.) to the bleeding scale established for this study the majority of patients would bleed 1-10ml/min so over 10 minutes that would be 10-100mls max. None of these patients even meet criteria of Class 1 of 750ml in the STS ATLS Trauma bleeding scale.

While we do not have this data available, we provide in the article the data for time to hemostasis: The time required to achieve hemostasis was lower in the AMP group (1.91±1.15 minutes) than in the SC group (2.28±2.09 minutes), although not significant (p=.309).

**3. The bleeding severity score use was only semi-quantitative. Providing a discussion of why a quantitative bleeding severity score was not employed, what such scores exist, and how not using one may affect results would be appropriate.**

For this study the investigators decided to devise a study specific bleeding severity score because in 2015 there were no validated quantitative bleeding scales published and peer reviewed. The only validated quantifiable bleeding chart available at the time was the STS ATLS Trauma bleeding scale from 2013.

There were bleeding scales used in many clinical trials. We acknowledge that they are

⦁	“Subjective” description of bleeding

⦁	Valid for investigators only within a particular clinical specialty

⦁	Valid for only a particular type of intervention

For example:

Oz et al used a bleeding scale in cardiovascular surgery in 2000 to describe the initial level of bleeding so as to evaluate the efficacy of a flowable hemostat.

The bleeding severity at each site was characterized as "oozing" or "heavy bleeding" (flowing or spurting). They considered success at 3 minutes and at 10 minutes by severity of bleeding. (Oz MC, Cosgrove DM 3rd, Badduke BR, Hill JD, et al. Controlled clinical trial of a novel hemostatic agent in cardiac surgery. The Fusion Matrix Study Group. Ann Thorac Surg. 2000 May;69(5):1376-82.)

Izzo et al used a bleeding scale for hepatic surgery in 2008 to measure and evaluate the efficacy of a hemostatic matrix before and after treatment.

A hemostatic matrix was applied directly to areas of bleeding. The severity of bleeding before and after application was graded on a 5-point scale (0 = no bleeding, 1 = oozing, 2 = moderate blood flow, 3 = heavy blood flow, 4 = spurting blood). The time to complete hemostasis was also recorded. After application of the gelatin matrix, bleeding severity was assessed at 1-min intervals until hemostasis was complete. (Izzo F, Di Giacomo R, Falco P, Piccirillo M, et al. Efficacy of a hemostatic matrix for the management of bleeding in patients undergoing liver resection: results from 237 cases. Curr Med Res Opin. 2008 Apr;24(4):1011-5.)

The first quantitative validated bleeding scale for intraoperative bleeding is the one published by Lewis KM et al. in 2017 (Lewis KM, Li Q, Jones DS, Corrales JD, Du H, Spiess PE, Lo Menzo E, DeAnda A Jr. Development and validation of an intraoperative bleeding severity scale for use in clinical studies of hemostatic agents. Surgery. 2017 Mar;161(3):771-781. doi: 10.1016/j.surg.2016.09.022.), but as previously mentioned this study started before it was published.

**4. Addition to the discussion of how electrocautery was the SC when AMP-SPH is approved for use when conventional treatments are ineffective or impractical. Explaining why it would not have been more appropriate to use another established hemostat (for example a flowable) as the SC in all SC subjects should be discussed.**

Electrocautery has long been recognized as a standard method for achieving hemostasis in various surgical procedures. Its widespread use and acceptance in clinical practice make it a relevant control for evaluating new hemostatic agents. Electrocautery is effective for controlling bleeding in many situations, particularly in cases of minor to moderate bleeding, and is often the first-line approach in surgical settings.

The primary aim of our study was to assess the efficacy of AMP-SPH in situations where conventional treatments may be insufficient. While AMP-SPH is approved for use when conventional methods are ineffective or impractical, it is essential to establish its effectiveness against a widely accepted standard. The choice of electrocautery as the control group reflects real-world clinical scenarios where surgeons often rely on this method. By comparing AMP-SPH to electrocautery, we can provide insights into its potential advantages in practice, particularly in complex cases where bleeding control is critical.

While flowable hemostatic agents are indeed established alternatives, they may not be universally applicable in all surgical contexts. The choice of hemostatic agent often depends on the specific surgical procedure, the nature of the bleeding, and the surgeon's preference. Additionally, many flowable hemostatic devices also contain thrombin making the device an “Active” hemostatic device which is in a different category than AMP-SPH which is considered a “Passive” hemostat. Electrocautery remains a versatile tool that can be employed in a variety of situations, making it a suitable control for our study.

We acknowledge the importance of exploring the comparative effectiveness of AMP-SPH against other established hemostatic agents in future studies. This could provide a more comprehensive understanding of its role in the surgical armamentarium and help refine guidelines for hemostatic management in various clinical settings.

**5. Reporting of the incidence of rebleeding after 10 minutes intraoperatively.**

In the AMP-SPH group, only 1 patient did not achieve hemostasis within 10 minutes. Bleeding was controlled after the 10 min by other methods (not described).

In the SC group, 3 patients did not achieve hemostasis within 10 minutes. Bleeding was controlled in 1 patient with AMP-SPH, in another with sutures, and in the last one by other methods not described by the investigator.

During this study, no other rebleeding events were described intraoperatively and no patients required reintervention for post- operative bleeding.

The following paragraph was added in the results section: “In the AMP-SPH group, hemostasis within 10 minutes was achieved in all but one patient; bleeding in this case was subsequently controlled by unspecified methods. In the SC group, three patients failed to achieve hemostasis within 10 minutes. Bleeding was controlled in these cases using AMP-SPH (n=1), sutures (n=1), and unspecified methods (n=1). No intraoperative rebleeding events or postoperative reinterventions for bleeding were reported during the study”.

**6. Explanation of what were the pre-analysis criteria for statistical non-inferiority.**

We acknowledge the study limitation of the lack of statistically valid sample size justification. This was added as a study limitation.

The current study was a Phase IV, observational (post-market) study designed to provide descriptive and exploratory data rather than confirmatory evidence. As stated in the study protocol, the sample size was not determined to achieve a specific statistical power to detect differences between the AMP-SPH and Standard of Care (SoC) patient populations. Instead, this study followed an observational, case-controlled design, in which patients were grouped based on treatment exposure rather than through true randomization, which is characteristic of experimental studies such as randomized controlled trials (RCTs). The AMP-SPH group represented the case study cohort, while the SoC group served as the control, allowing for comparative assessment of specific clinical characteristics between the two cohorts.

Given the exploratory nature of this study, our primary objective was to gather preliminary evidence rather than to definitively establish non-inferiority. Although prospective sample size calculation is the preferred methodological approach, as the reviewer correctly noted, a post-hoc analysis was conducted to estimate the number of patients required to detect significant differences based on the observed results. This analysis provided valuable insights into the potential adequacy of the study’s sample size, reinforcing the descriptive and hypothesis-generating role of this investigation.

The following paragraph was added as a study limitation: “Another important limitation of this study is the lack of a statistically valid sample size justification. While a prospective sample size calculation would have been the ideal approach, a post-hoc analysis was performed to estimate the number of patients required to detect significant differences based on the observed results. With an alpha risk of 0.05 in a two-sided test and sample sizes of 44 patients in the AMP-SPH group and 46 in the SC group, the statistical power to detect the observed differences as significant was only 16%. Furthermore, to achieve a power of 80% with an alpha of 0.05, a total of 353 patients per group would have been required for the observed differences in the proportion of patients achieving hemostasis within 10 minutes to reach statistical significance.

**7. Clarification as to why all subjects were not able to be included in the final analysis.**

The Per Protocol (PP) population includes all subjects who were randomized and

treated with PerClot, and had no major protocol deviations, where major protocol

deviations are defined as:

- Failure to meet any preoperative inclusion/exclusion criteria;
- Failure to meet any intraoperative inclusion/exclusion criteria; or
- Any informed consent violation.

The patients excluded from the PP population had major protocol deviations. Four (4) deviations were determined to be subjects not meeting the Inclusion Criteria or meeting the Exclusion Criteria of the study and the remaining deviations were Informed Consent Form deviations. All subjects were consented to the study, but there were various deviations for the method and/or process of consenting, which included signing the wrong version of the informed consent, the incorrect person dating the consent, or an unauthorized Investigator signing the consent. It was verified that the changes in the different versions of the informed consent were minor and would not have impacted the safety of the patient.

**8. Discussion of why there was a trend toward more hematomas in the AMP-SPH group.**

In our study, the most commonly reported AE in the AMP group was hematoma, which occurred in 5 cases: 2 cases of pelvic hematoma (one with vaginal spotting); 1 Douglas hematoma; 1 vaginal cuff hematoma; and 1 cupula hematoma. There was one occurrence of hematoma in the SC group. This translated into an incidence of 11.4% in the AMP group and 2.2% in the SC group, but despite this, the difference was not statistically significant.

The following paragraph was added in the discussion section: “The incidence of hematoma formation following pelvic gynecological surgery is highly variable, ranging from 25% to 98% [30]. Established risk factors include the type of surgery, patient comorbidities, surgical technique, oncologic resections, difficult anatomical exposure, prior surgeries, recurrent tumors, and previous radiation therapy [31]. Although these factors may have influenced the observed, non-significant differences in hematoma rates between the study groups, no differences in the distribution of these risk factors were identified”.

**9. In the abstract as well as on page 2 line 44-45 and page 16, line 357-9, the word “observed” could be added for the comparison on amount of blood transfusions being less in the AMP-SPH group as this difference was not statistically significant.**

Both changes were done.

Abstract: The sentence was changed to: “A higher proportion of patients in the SC group (17.9%) was observed to require blood products compared to those in the AMP group (4.8%)”.

Lines 357-9: The sentence was changed to: "Although a higher proportion of patients in the SC group (17.9%) was observed to require blood products compared to the AMP group (4.8%), this difference did not reach statistical significance."

**Reviewer #4:**

**The authors of the manuscript have properly addressed all the Reviewer´s comments and concerns. Congratulations.**

Thank you very much indeed for the comment, we highly appreciate it.
