# Supplementary material for: Effectiveness and safety of an absorbable modified polymer starch powder hemostat versus usual care in gynecology procedures: A prospective, multi-center, and randomized study
Source: PLoS One. 2025 Sep 11;20(9):e0331376. doi: 10.1371/journal.pone.0331376 (PMC12425258; doi:10.1371/journal.pone.0331376)
Supplement: Attachment — Submitted filename: Rebuttal letter PerClot PlosOne R3 1.0.docx [file pone.0331376.s008.docx]

Response to the reviewers, manuscript PONE-D-25-02293R2, entitled

“Effectiveness and safety of an absorbable modified polymer starch powder hemostat versus usual care in gynecology procedures: A prospective, multi-center, and randomized study”.

Dear Editor-in-Chief, I have considered the comments made by the reviewers and I hope that the paper improves greatly thanks to these comments.

I am sending you the revised manuscript and the rebuttal letter providing a point-by-point response to each of the numbered reviewer comments.

**# Review Comments to the Author
Reviewer #1: (No Response)**

**Reviewer #2: WELL DESIGNED AND WRITTEN STUDY REGARDING HEMOSTATIC AGENTS WHICH DO HAVE PROFOUND IMPACT IN OUR DAILY PRACTICE**

Thank you very much indeed for the comment, we highly appreciate it.

**Reviewer #3: In this second revision of the submission, Dr. Garcia-Tejedor and colleagues have contrasted the use of absorbable modified polymer starch powder hemostat (AMP-SPH) compared to standard of care (SC).
The submission has been significantly improved.
An additional modification would further strengthen the submission.
1. Provide the time span over which patient enrollment occurred in terms of chronological dates.**

The following paragraph was added in the results section: “A total of 90 patients were enrolled across two study sites between November 2015 and July 2017. Screening concluded in July 2017 upon reaching the target enrollment. The study duration was 21 months, from the first patient’s initial visit to the last patient’s final visit. Of the 90 enrolled participants, 44 (48.9%) were assigned to the AMP group and 46 (51.1%) to the SC group (Figure 1).”

**Reviewer #4: The authors of the manuscript have addressed all possible reviewers concerns. Nothing else to comment**

Thank you very much indeed for the comment, we highly appreciate it.
